# Supplementary figures and images for: Explosive and implosive root concepts: An analysis of music moods rooted by two influential rap artists
Source: PLoS One. 2022 Jul 1;17(7):e0270648. doi: 10.1371/journal.pone.0270648 (PMC9249228; doi:10.1371/journal.pone.0270648)

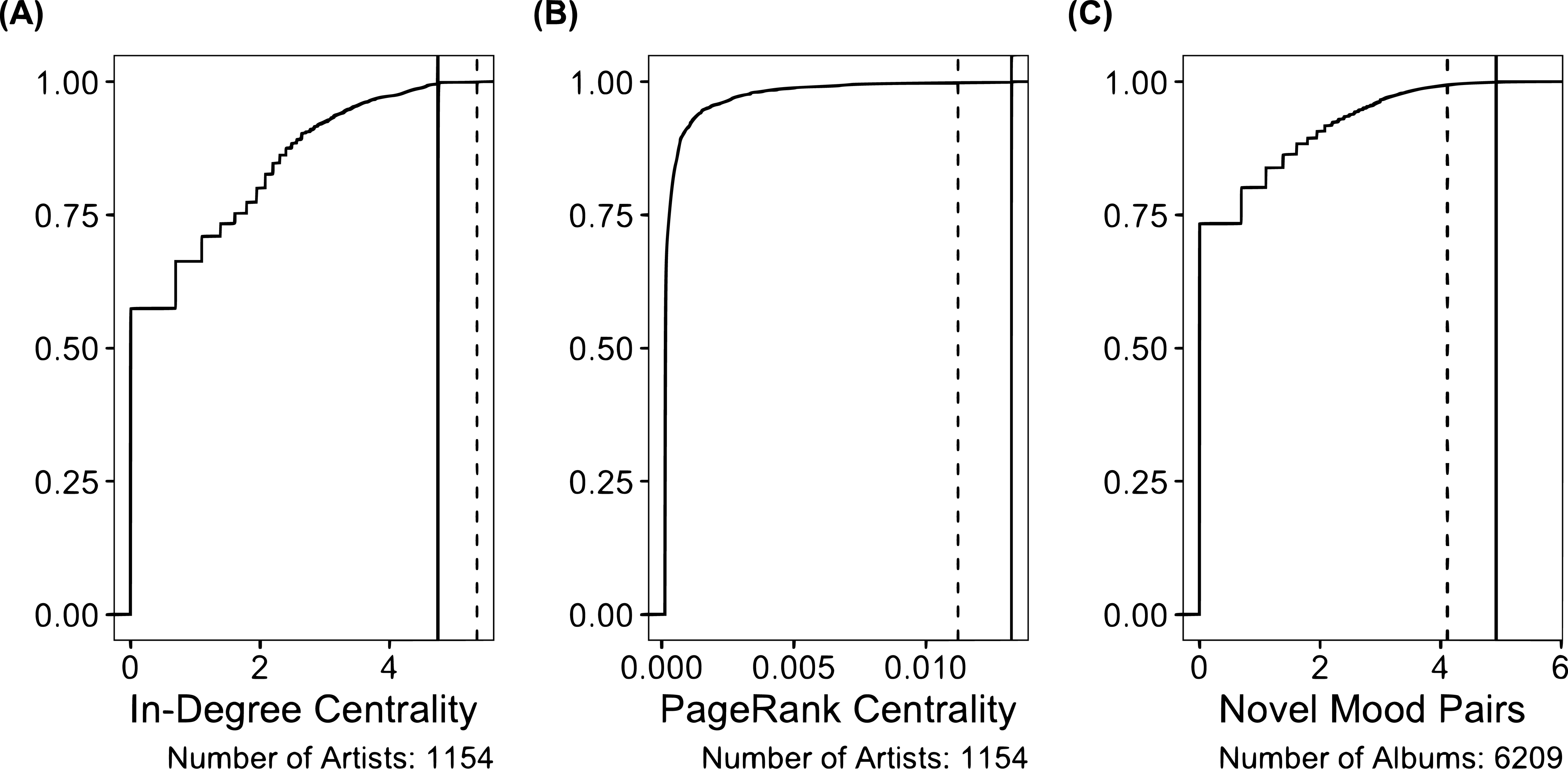

Supplement: S1 Fig — To ensure that Run-D.M.C. and N.W.A are uniquely different from other artists, for the data on artists in our sample, we plotted the cumulative population-level distributions. The x-axis of panels (A) and (B) denote in-degree centrality and PageRank centrality, respectively, as influence indicators. The x-axis of panel (C) is the number of novel mood pairs as an indicator of novelty. The vertical solid and dotted lines indicate the data for Run-D.M.C. and N.W.A, respectively. They are both located at the right end of the cumulative distributions, suggesting their exceptional influence and novelty. The in-degree centrality, PageRank, and novelty scores for Run-D.M.C. are 0.998, 1.000, and 0.999, respectively, and those for N.W.A are 1.000, 0.998, and 0.994, respectively. The data suggest the two artists have extreme scores. (TIF) [file pone.0270648.s001.tif]

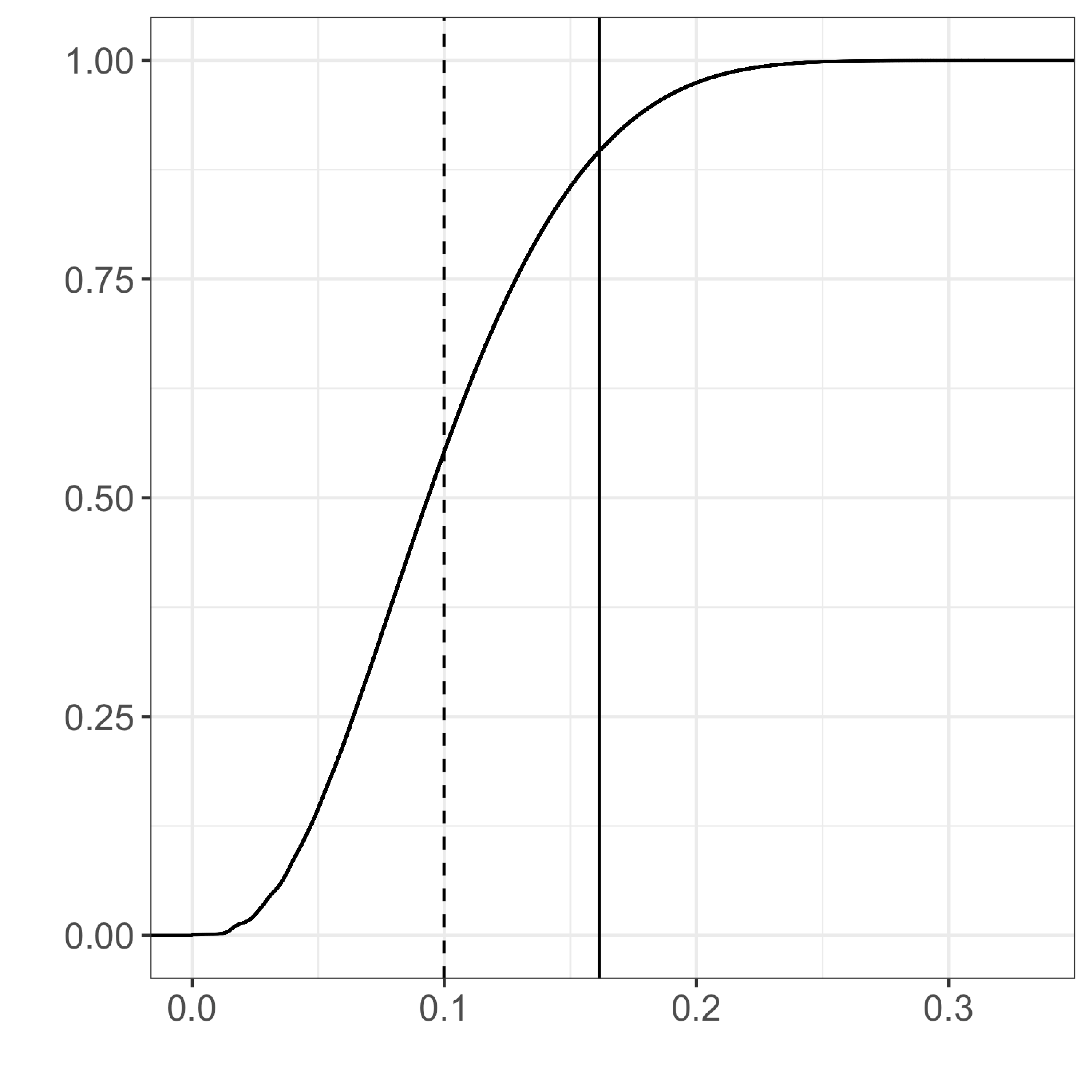

Supplement: S2 Fig — The Euclidean distance between Run-D.M.C.’s vector and N.W.A’s vector is that its location is at the 0.90 percentile in the distribution of all possible pairs of albums’ distances. The solid vertical line indicates the root’s distance. The dotted line represents the mean distance. (TIF) [file pone.0270648.s002.tif]

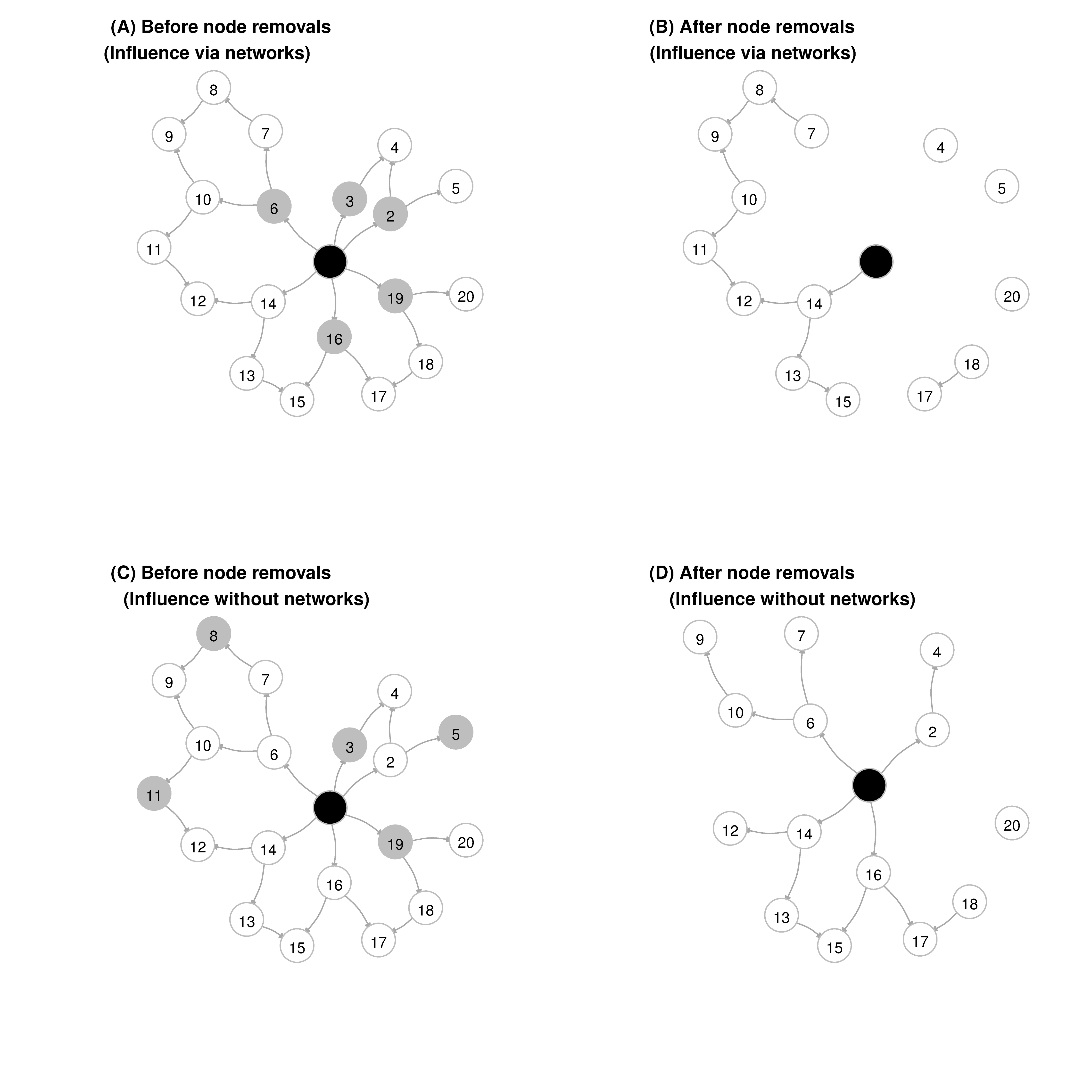

Supplement: S3 Fig — Dots and edges represent artists and the directional influences between them. The black dots in the center are root concept creators. The gray dots are artists who work with the collaborators of root concept creators. The white dots are other artists. These diagrams illustrate changes in the overall network structures after node removal. The impact of removing nodes that work with root concept creators’ collaborators is greater in network a, where root concept creators’ influences and network contagions are closely related. The diagrams suggest that changes can be captured best with the reachability of the remaining nodes. (TIF) [file pone.0270648.s003.tif]
