# Supplementary material for: Explosive and implosive root concepts: An analysis of music moods rooted by two influential rap artists
Source: PLoS One. 2022 Jul 1;17(7):e0270648. doi: 10.1371/journal.pone.0270648 (PMC9249228; doi:10.1371/journal.pone.0270648)
Supplement: S1 Table — (PDF) [file pone.0270648.s004.pdf]

Variables	Unit	N	Mean	SD	Min	Max	Run-D.M.C.	N.W.A
Number of moods	Album	6,209	11.90	5.49	2	44	26 (0.99)	28 (0.99)
Number of mood pairs	Album	6,209	80.20	75.73	1	946	325 (0.98)	378 (0.99)
Number of novel mood pairs	Album	6,209	2.61	10.36	0	313	136 (1.00)	60 (0.99)
In-degree centrality	Artist	1,154	5.71	16.17	0	210	114 (1.00)	210 (1.00)
PageRank centrality	Artist	1,154	0.00	0.00	0	0.01	0.01 (1.00)	0.01 (1.00)

The numbers in parentheses indicate the percentile of the root concept scores.
