# Supplementary material for: Explosive and implosive root concepts: An analysis of music moods rooted by two influential rap artists
Source: PLoS One. 2022 Jul 1;17(7):e0270648. doi: 10.1371/journal.pone.0270648 (PMC9249228; doi:10.1371/journal.pone.0270648)
Supplement: S2 Table — (PDF) [file pone.0270648.s005.pdf]

| | **Variables** | **Mean** | **SD** | **Min** | **Max** |
|---|---|---|---|---|---|
| 1 | Combined elements | 55.80 | 39.70 | 1 | 205 |
| 2 | Element diversity | 0.95 | 0.08 | 0 | 0.99 |
| 3 | Combinatory strength | 2.73 | 3.32 | 1 | 41.40 |
| 4 | Run-D.M.C. dummies | 0.16 | 0.37 | 0 | 1 |
| 5 | N.W.A dummies | 0.17 | 0.38 | 0 | 1 |
| 6 | Mood age | 13.50 | 8.35 | 1 | 38 |
| 7 | Mood popularity | 17.20 | 37.70 | 0 | 519 |
| 8 | Total number of moods | 173.00 | 45.00 | 94 | 242 |
| 9 | Total number of released albums | 270.00 | 189.00 | 40 | 772 |

| | 1 | 2 | 3 | 4 | 5 | 6 | 7 | 8 | 9 |
|---|---|---|---|---|---|---|---|---|---|
| 1. | 1 | | | | | | | | |
| 2. | 0.41 | 1 | | | | | | | |
| 3. | 0.67 | 0.16 | 1 | | | | | | |
| 4. | 0.40 | 0.13 | 0.44 | 1 | | | | | |
| 5. | 0.31 | 0.12 | 0.41 | 0.34 | 1 | | | | |
| 6. | 0.77 | 0.30 | 0.53 | 0.36 | 0.35 | 1 | | | |
| 7. | 0.66 | 0.15 | 0.91 | 0.43 | 0.36 | 0.54 | 1 | | |
| 8. | 0.36 | 0.19 | 0.09 | -0.11 | -0.11 | 0.39 | 0.16 | 1 | |
| 9. | 0.36 | 0.12 | 0.39 | -0.05 | -0.05 | 0.27 | 0.30 | 0.31 | 1 |
