# Supplementary material for: Explosive and implosive root concepts: An analysis of music moods rooted by two influential rap artists
Source: PLoS One. 2022 Jul 1;17(7):e0270648. doi: 10.1371/journal.pone.0270648 (PMC9249228; doi:10.1371/journal.pone.0270648)
Supplement: S3 Table — (PDF) [file pone.0270648.s006.pdf]

	1	2	3
	**Combined elements**	**Element diversity**	**Combinatory strength**
Run-D.M.C. dummies	0.28***	0.17***	0.14***
	(0.07)	(0.04)	(0.03)
N.W.A dummies	−0.01	0.12**	0.23***
	(0.06)	(0.04)	(0.03)
Mood age	0.53***	0.26***	0.05***
	(0.03)	(0.03)	(0.01)
Mood popularity	0.28***	−0.07***	0.79***
	(0.04)	(0.01)	(0.02)
Total number of moods	0.25***	0.08*	−0.07***
	(0.03)	(0.04)	(0.02)
Total number of released albums	0.24***	0.06***	0.31***
	(0.02)	(0.02)	(0.03)
Constant	−0.22***	0.01	−0.03
	(0.05)	(0.06)	(0.03)
5-year cohort dummies	Yes	Yes	Yes
Observations	4,040	4,040	4,040

Robust standard errors are in parentheses. Dependent variables are scaled.

*$p < 0.05$, **$p < 0.01$, ***$p < 0.001$
