# Supplementary material for: Explosive and implosive root concepts: An analysis of music moods rooted by two influential rap artists
Source: PLoS One. 2022 Jul 1;17(7):e0270648. doi: 10.1371/journal.pone.0270648 (PMC9249228; doi:10.1371/journal.pone.0270648)
Supplement: S4 Table — (PDF) [file pone.0270648.s007.pdf]

	**Variables**	**Mean**	**SD**	**Min**	**Max**
1	Similarity (Run-D.M.C.)	0.97	0.02	0.82	1.00
2	Similarity (N.W.A)	0.94	0.04	0.77	1.00
3	Working with Run-D.M.C.'s collaborators	0.03	0.17	0	1
4	Working with N.W.A's collaborators	0.04	0.20	0	1
5	Gang affiliation	0.02	0.15	0	1
6	Affluent production resources	1.49	2.67	0	28
7	Location NY	0.05	0.22	0	1
8	Location CA	0.02	0.14	0	1
9	Previous releases	2.73	2.89	1	24
10	Team size	21.80	20.60	1	187

	1	2	3	4	5	6	7	8	9	10
1.	1									
2.	0.39	1								
3.	0.02	-0.01	1							
4.	0.03	0.09	0.04	1						
5.	0.00	0.08	0.06	0.14	1					
6.	-0.09	-0.19	0.08	0.03	0.02	1				
7.	0.01	0.10	0.13	0.02	0.00	0.04	1			
8.	0.04	0.05	-0.01	0.14	0.10	-0.01	-0.03	1		
9.	0.01	0.07	0.03	0.06	0.14	0.05	0.12	0.13	1	
10.	0.06	-0.06	0.20	0.16	0.07	0.51	0.15	0.04	0.11	1
