# Supplementary material for: Explosive and implosive root concepts: An analysis of music moods rooted by two influential rap artists
Source: PLoS One. 2022 Jul 1;17(7):e0270648. doi: 10.1371/journal.pone.0270648 (PMC9249228; doi:10.1371/journal.pone.0270648)
Supplement: S5 Table — (PDF) [file pone.0270648.s008.pdf]

	1	2
	**Expressing Run-D.M.C.-like Moods OLS**	**Expressing N.W.A-like Moods OLS**
Working with Run-D.M.C.'s collaborators	−0.07	−0.13
	(0.07)	(0.07)
Working with N.W.A's collaborators	−0.01	0.37***
	(0.05)	(0.05)
Gang affiliation	−0.09	0.39***
	(0.07)	(0.07)
Atypical production resources	−0.15***	−0.25***
	(0.03)	(0.03)
Location NY	−0.07	0.48***
	(0.04)	(0.05)
Location CA	0.26***	0.20**
	(0.05)	(0.07)
Previous releases	0.01**	0.03***
	(0.00)	(0.00)
Team size	0.01***	−0.00
	(0.00)	(0.00)
Constant	0.13**	0.00
	(0.05)	(0.05)
5-year cohort dummies	Yes	Yes
Observations	6,111	6,111
R^2	0.063	0.081

Robust standard errors are in parentheses. Dependent variables are scaled.

* $p < 0.05$, ** $p < 0.01$, *** $p < 0.001$.
